# Comparison of Cone Mosaic Metrics From Images Acquired With the SPECTRALIS High Magnification Module and Adaptive Optics Scanning Light Ophthalmoscopy

**DOI:** 10.1167/tvst.11.5.19

**Published:** 2022-05-18

**Authors:** Niamh Wynne, Heather Heitkotter, Erica N. Woertz, Robert F. Cooper, Joseph Carroll

**Affiliations:** 1 Department of Ophthalmology and Visual Sciences, Medical College of Wisconsin, Milwaukee, WI, USA; 2 Department of Cell Biology, Neurobiology and Anatomy, Medical College of Wisconsin, Milwaukee, WI, USA; 3 School of Medicine, Medical College of Wisconsin, Milwaukee, WI, USA; 4 Joint Department of Biomedical Engineering, Marquette University and Medical College of Wisconsin, Milwaukee, WI, USA

**Keywords:** retinal imaging, adaptive optics, quantitative metrics

## Abstract

**Purpose:**

To compare cone mosaic metrics derived from adaptive optics scanning light ophthalmoscopy (AOSLO) images with those derived from Heidelberg Engineering SPECTRALIS High Magnification Module (HMM) images.

**Methods:**

Participants with contiguous cone mosaics had HMM imaging performed at locations superior and temporal to the fovea. These images were registered and averaged offline and then aligned to split-detection AOSLO images; 200 × 200-µm regions of interest were extracted from both modalities. Cones were semi-automatically identified by two graders to provide estimates of cone density and spacing.

**Results:**

Thirty participants with contiguous cone mosaics were imaged (10 males, 20 females; age range, 11–67 years). Image quality varied, and 80% of our participants had analyzable HMM images. The intergrader intraclass correlation coefficients for cone metrics were good for both modalities (0.688–0.757 for HMM; 0.805–0.836 for AOSLO). Cone density estimates from HMM images were lower by 2661 cones/mm^2^ (24.1%) on average compared to AOSLO-derived estimates. Accordingly, HMM estimates of cone spacing were increased on average compared to AOSLO.

**Conclusions:**

The cone mosaic can be visualized in vivo using the SPECTRALIS HMM, although image quality is variable and imaging is not successful in every individual. Metrics extracted from HMM images can differ from those from AOSLO, although excellent agreement is possible in individuals with excellent optical quality and precise co-registration between modalities.

**Translational Relevance:**

Emerging non-adaptive optics-based photoreceptor imaging is more clinically accessible than adaptive optics techniques and has potential to expand high-resolution imaging in a clinical environment.

## Introduction

Single-cell resolution of the photoreceptor mosaic is routinely obtained through the use of various adaptive optics (AO)-based retinal imaging modalities.^1^^–^^3^ These images enable extraction of quantitative metrics of the cone mosaic^4^^,^^5^ that are comparable to those obtained from histological samples.^6^^–^^8^ Such images allow more sensitive assessment of pathology when compared with conventional clinical measures of retinal structure and function.^9^ For example, defects in the photoreceptor mosaic have been documented with adaptive optics scanning light ophthalmoscopy (AOSLO), even in retinas with normal optical coherence tomography (OCT) findings.^10^^,^^11^ Similarly, AOSLO can detect diffuse cone loss even when visual acuity and sensitivity remain within normal limits.^12^ Despite the capabilities of AO-enhanced ophthalmoscopy and its potential clinical applications, high costs and limited availability of imaging devices remain barriers to widespread clinical use. A flood-illumination AO system (rtx1) is commercially available from Imagine Eyes, Inc. (Orsay, France) and can be used to quantitatively assess extrafoveal cones^13^^–^^22^; however, it cannot resolve foveal cones or rods and is not currently 510(k) cleared by the U.S. Food and Drug Administration.^16^^,^^23^ Thus, the majority of translational AO imaging remains limited to costly custom-built devices.

A middle ground is emerging, with lower costs and the potential for greater clinical accessibility, offering the opportunity to significantly expand the options for high-resolution imaging of the cone mosaic. Although it has been known for over two decades that images of the parafoveal cone mosaic can be acquired without AO (especially in eyes with good optical quality),^24^^,^^25^ there has been renewed interest in this approach. A number of groups have used full-field or transverse OCT imaging to resolve the parafoveal cone mosaic,^26^^,^^27^ with further improvement resulting from careful correction of axial eye motion.^28^^,^^29^ In addition, imaging of the cone mosaic has been demonstrated with advanced scanning laser ophthalmoscope systems,^30^^–^^33^ including one incorporated into a handheld probe.^34^ Among these high-definition ocular imaging devices that function without AO is the High Magnification Module (HMM) for the SPECTRALIS system (Heidelberg Engineering, Heidelberg, Germany), which is a commercially available lens and software module that functions with their standard clinical operating system. This near-infrared reflectance modality reveals microstructures resembling the cone mosaic and has been used to qualitatively assess the cone mosaic in multiple retinal pathologies.^35^^–^^37^

Quantitative metrics of the photoreceptor mosaic in HMM images of individuals with normal visual acuity have been consistent with reported AO and histological values.^38^^–^^40^ Following initial reports suggesting poor interobserver repeatability of cone density on the HMM,^38^ there has been an interest in exploring the utility of automated algorithms for cone identification in HMM images.^39^^,^^40^ Our present study utilized an established semi-automated cone-identification algorithm across both modalities; the primary purpose was to compare quantitative metrics between AOSLO and HMM in the same individuals.

## Methods

### Participants

This study was approved by the institutional review board (IRB) at the Medical College of Wisconsin (PRO00030741) and conformed to the tenets of the Declaration of Helsinki. Written informed consent was obtained from all participants prior to participation. We included 27 individuals with normal vision and three individuals diagnosed with oculocutaneous albinism; two previously reported siblings (JC_0492 and JC_0493) with two pathogenic mutations in *TYR*—c.1147G>A (p.383Asp>Asn) and c.1217C>T (p.406Pro>Leu)^41^; and one (JC_12277) who had multiple albinism-related sequence variants—*OCA2*, c.1327G>A (p.Val443Ile); *TYR*, c823G>T (p.Val275Phe); and *TYR*, c.575C>A (p.Ser192Tyr). The average age ± SD of participants was 29 ± 13.1 years, with a range of 11 to 67 years. There were 10 males and 20 females. All participants had axial length measurements acquired with an IOLMaster (Carl Zeiss Meditec, Dublin, CA) at the time of imaging for subsequent scaling of images. HMM imaging was carried out during the same visit as AOSLO imaging for 17 participants; the remaining 13 participants had HMM imaging performed between 6 months before and 18 months after their AOSLO imaging.

### HMM Image Acquisition and Processing

**Figure 1.**
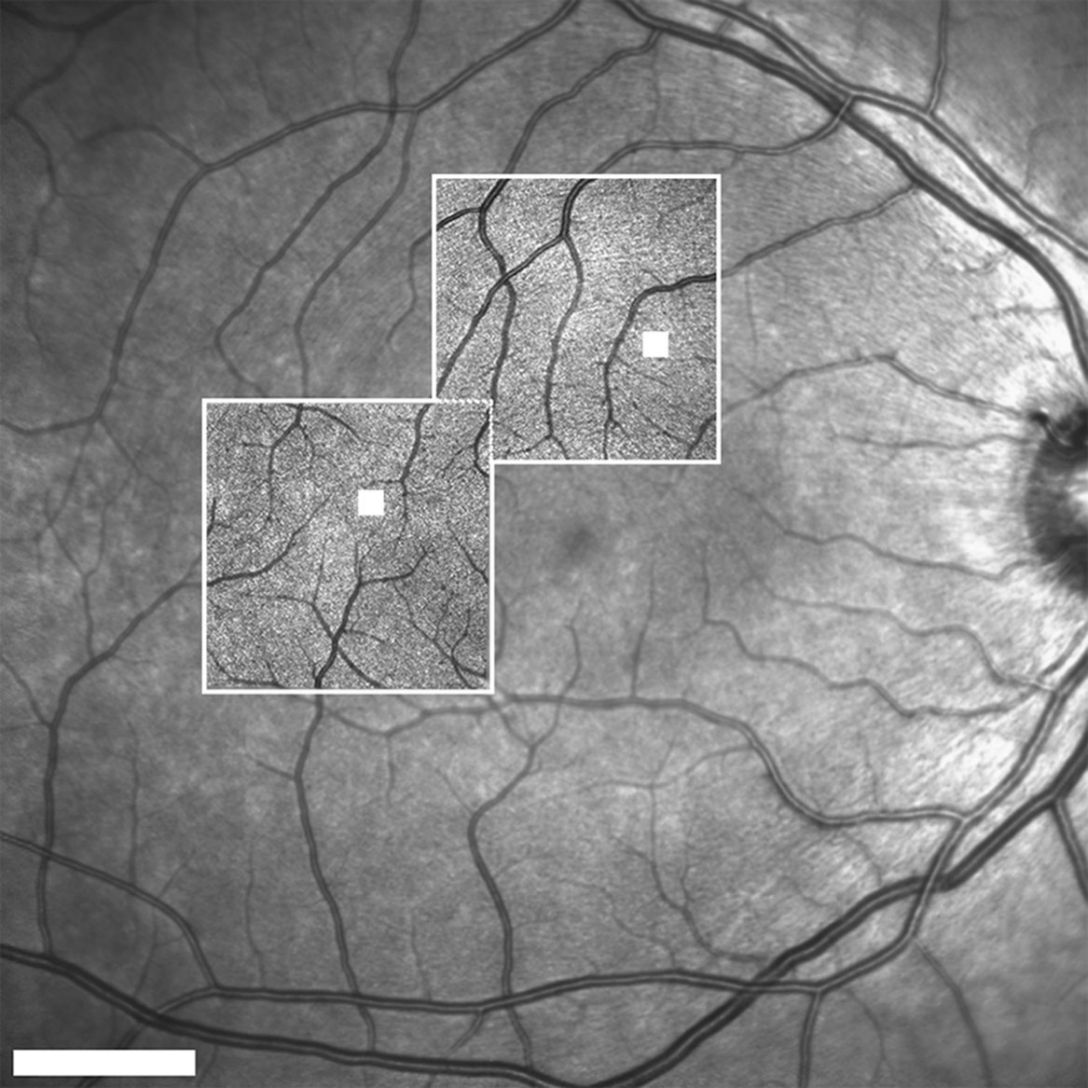
A 30° near-infrared reflectance image of a 23-year-old male participant. The 8° HMM images at superior and temporal locations are outlined in *white*. Within each HMM image, a 200 × 200-µm region of interest is indicated by a *filled-in white square*. *Scale bar*: 5°.

HMM near-infrared reflectance retinal images were acquired using the Heidelberg SPECTRALIS confocal scanning laser ophthalmoscopy system (including Heidelberg HRA SPECTRALIS hardware and Heidelberg Eye Explorer 1.10.4.0 software). Participants were imaged with undilated pupils. Use of a light directed toward the fellow eye induced the consensual pupillary reflex and enhanced pupil constriction, improving image quality in three individuals; however, in the remainder this approach did not perceptibly impact image quality. Although imaging with corrective lenses has been reported to improve image quality (Bartsch DUG, et al. *IOVS*. 2021;62:ARVO E-Abstract 24),^37^ we did not use this technique in order to minimize the introduction of alterations in retinal magnification (and thus image scale).^42^ See Supplementary Figure S1 for demonstration of scale change with refractive correction. Each subject had one eye imaged at two locations, one superior and one temporal to the fovea, using the device's internal fixation target (Fig. 1). The high-speed setting, which obtained images at 8.8 frames per second, was chosen over the high-resolution mode, which captures images at a rate of 4.7 frames per second, with the intention of minimizing image distortions^43^ across individuals with varying eye movements. The sensitivity and focus were manually adjusted to subjectively optimize visualization of the photoreceptors.

**Figure 2.**
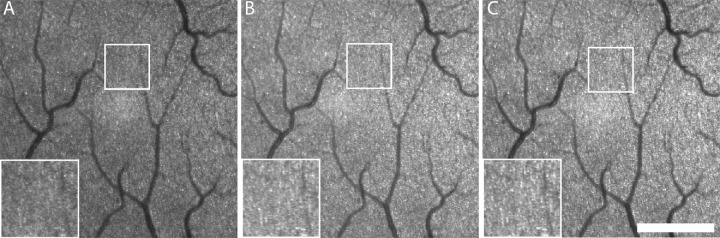
Comparison of raw HMM image quality with averaging and postprocessing. Images of the same temporal ROI in a 31-year-old male participant with normal vision: (A) single frame; (B) real-time averaging of 15 frames using the ART mode on the SPECTRALIS device; and (C) extra offline averaging of six ART averaged images. A 400-µm region is highlighted in each image, with a zoomed-in view of the same region in the *bottom left* of each panel. *Scale bar*: 700 µm. See Supplementary Video S1 for overlaid images.

The number of images captured at each location varied depending on the ease of visualization of the photoreceptors and the homogeneity of image quality across the 8° field of view. The Automatic Real-Time Tracking (ART) mode was used for each image, where each individual image captured was an average of 15 frames. Averaging of a greater number of frames in ART mode led to increased blur due to accumulating registration errors, especially in individuals with greater movement due to age or nystagmus. Across all participants, between five and 50 averaged images were collected at each location imaged (average = 14.9). Higher numbers of images were collected in individuals with poorer visualization of structures and less uniform image quality to allow for later offline registration. Before exporting from the device, Heidelberg Eye Explorer 1.10.4.0 was used to adjust the brightness and contrast of each image to optimize photoreceptor visualization. The HMM images from each location within a subject were then aligned using an affine registration model (i2k Retina; DualAlign LLC, Clifton Park, NY). Finally, the aligned images were reviewed by one grader (NW), and images with low contrast of reflective structures and/or less uniform image quality were removed, with the remaining images averaged using the Image J Z-project plugin (Fig. 2).^44^

### AOSLO Image Acquisition and Processing

Thirteen participants had AOSLO images acquired prospectively during the same visit as HMM. AOSLO images not collected at the time of HMM imaging were accessed from an IRB-approved image bank housed at the Medical College of Wisconsin (PRO 30741). For each AOSLO imaging session, the subject's cranial movement was minimized using a dental impression on a bite bar. Images were simultaneously collected using the confocal and non-confocal (split detection) modalities of the system. Fields of view used for imaging ranged from 1° to 1.5°, extending from the fovea at 0.5° intervals to capture a contiguous temporal and superior strip extending out to 10° to 12° eccentricity. Imaging light of 775-nm or 790-nm wavelength was used to acquire sequences of 150 to 250 frames at each location. Individuals with nystagmus and other factors compromising image quality had more frames collected, whereas fewer frames were sufficient for those with stable fixation and good image quality. The raw frames from each image sequence were corrected for sinusoidal distortions and strip-registered to an automatically selected reference frame as previously described.^45^^,^^46^ Between 50 and 80 frames were then averaged to produce a single image with a high signal-to-noise ratio. As with number of frames collected, the number of frames averaged depended on the signal-to-noise ratio in the respective images. Further distortion was removed from the resultant TIFF image using “de-warping” software (https://github.com/OCVL/Eye-Motion-Repair). This software works by calculating the median (*x*, *y*) shift observed at each row of the registered image from the registration shift in each frame contributing to that image; it then “de-warps” the registered image using these median shifts, assuming random eye movement.^47^ The spatially co-registered confocal and split detection images were semi-automatically montaged using a multimodal montaging algorithm, and images from different fields of view were resampled to a common scale using Photoshop CS6 (Adobe, San Jose, CA).^48^

### Scale Calculation

IOLMaster measurements of axial length were taken at the time of imaging with each device, and contemporaneous measurements to each imaging session were used for calculations. The HMM linear scale (µm/pixel) was estimated according to the following equation:

$$\begin{array}{c} \mathrm{HMM}\;\mathrm{Scale}=\frac{\theta }{I_{s}}\mathrm{RMF}\left(\frac{\mathrm{AL}}{24}\right) \end{array}$$

where θ represents the scan size of the HMM image (degrees), *I_s_* represents the number of pixels in the raw HMM image, RMF represents the assumed retinal magnification factor (291 µm/deg) of an eye with a 24.0-mm axial length,^49^ and AL is the axial length of the subject (mm). The linear scale (µm/pixel) of the AOSLO images for a given eye was estimated by using the following equation:

$$\begin{array}{c} \mathrm{AOSLO}\;\mathrm{Scale}=\frac{T}{f_{1}T_{s}}\left(\frac{180}{\pi }\right)\mathrm{RMF}\left(\frac{\mathrm{AL}}{24}\right) \end{array}$$

where *T* represents the periodicity of a Ronchi ruling (µm/cycles), *f*_1_ represents the focal length of the model eye in our system (µm), *T_s_* represents the sampling period of the lines in the model eye image of the Ronchi ruling (pixels/cycle), RMF represents the assumed retinal magnification factor (291 µm/deg) of an eye with a 24.0-mm axial length,^49^ and AL is the axial length of the subject (mm).

### AOSLO and HMM Image Analysis

Given the non-uniform appearance of the photoreceptor mosaic in HMM images, both modalities were examined to identify areas of overlapping high-quality structural images. We then used Mosaic Analytics^5^ (Translational Imaging Innovations; Hickory, NC) to extract a 200 × 200-µm region of interest (ROI) from the AOSLO image in an area of overlap with the HMM image. An image of the overlaid ROI marker on the confocal AOSLO montage was coarsely scaled and aligned to the corresponding HMM image. This approximate marker was then used to position the ROI within the HMM image in Mosaic Analytics, ensuring co-localization of the ROIs between the modalities. Confocal AOSLO was used for alignment for its similar contrast of large vascular structures to HMM, whereas split-detection AOSLO ROIs were extracted for unambiguous cone identification.

ROIs were masked and cones were semi-automatically identified in all ROIs by two independent graders (NW, JC) using Mosaic Analytics.^5^ Bound cone density, nearest neighbor distance (NND), and intercell distance (ICD) were calculated from the respective coordinates for each ROI using a custom MATLAB script (MathWorks, Natick, MA).^5^

### Statistical Analysis

Based on the average cone density ± SD at 6° eccentricity, 15,528 ± 1808 cones/mm^2^ (derived from available AO literature^5^^,^^15^), and a sample size of 48 analyzable ROIs, this study was powered to detect a density difference of 6.75% between devices. This effect size was chosen based on previous reports of estimates of cone metric repeatability from AOSLO images of 2% to 10% (under changing conditions of the observer, imaging and sampling protocols, and imaging session).^50^^–^^52^

Intraclass correlation coefficients (ICCs) of interobserver density NND and ICD measurements for both AOSLO and HMM modalities were calculated using the RStudio 1.3.1093 ICCest function from the ICC package version 2.3.0 (R Foundation for Statistical Computing, Vienna, Austria). Bland–Altman analyses were conducted using Excel (Microsoft Corporation, Redmond, WA) to compare both interobserver and inter-method differences.^53^ In order to examine for an effect of the time between imaging sessions and retinal eccentricity on estimates, linear regressions were performed using Prism 9.0.0 (GraphPad Software, San Diego, CA) to compare the difference in average cone metrics between modalities to the time elapsed between imaging sessions (months) and retinal eccentricities (degrees).

## Results

**Figure 3.**
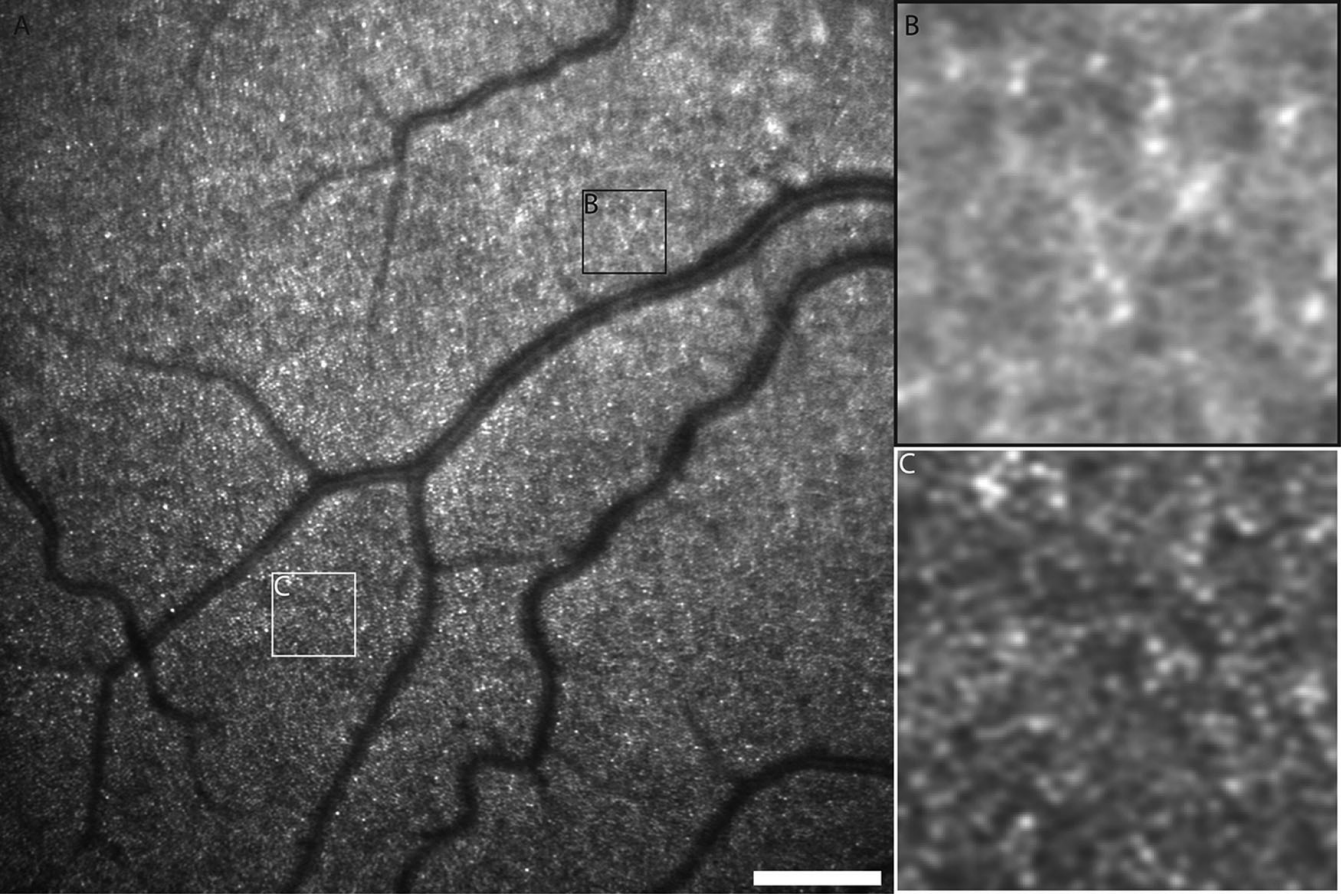
Demonstrating variable photoreceptor structure visibility across HMM images. HMM images of a 30-year-old male participant with normal vision: (A) HMM image demonstrating a 7° field of view of the superior retina. *Scale bar*: 1°. (B) Region with poor visibility of photoreceptor structure from within this image. (C) Region of particularly good visibility of photoreceptor structure from within the same image. The locations of these 200 × 200-µm regions are outlined in panel A.

Of 30 participants with contiguous mosaics imaged on HMM and AOSLO, 24 had analyzable HMM images, representing an 80% success rate. A combination of factors contributed to failure in the six participants with unanalyzable images: refractive errors, young age prohibiting collection of sufficient images, poor fixation, and nystagmus. The average ± SD axial length of participants was 24.31 ± 1.31 mm, with a range of 21.37 to 27.34 mm. Image quality varied across participants, between superior and temporal locations in the same individuals and across regions within individual 8° HMM images (Fig. 3). Visualization of the photoreceptor mosaic was not uniform across the entire field of view for any participant. A common feature observed to varying degrees during HMM imaging of all participants was an intermittent central, hyperreflective optical artifact that partially obscured the view of underlying photoreceptors. This artifact has been reported previously^37^^–^^39^ and is attributed by the Heidelberg operating manual to reflections from the internal optical surfaces of the HMM lens. The intensity of this artifact on the images could be minimized by manipulating the *z*-position (working distance), sensitivity, and use of artificial tears, although it was still present even after processing in 47.9% of HMM images. Images from all participants with analyzable images, although not equal in quality, were included for analysis. For superior ROIs, the average ± SD eccentricity was 6.7° ± 1.6° with a range of 3.0° to 10.0°; for temporal ROIs, the average eccentricity ± SD was 5.7° ± 1.7°, with a range of 2.9° to 9.1°.

**Figure 4.**
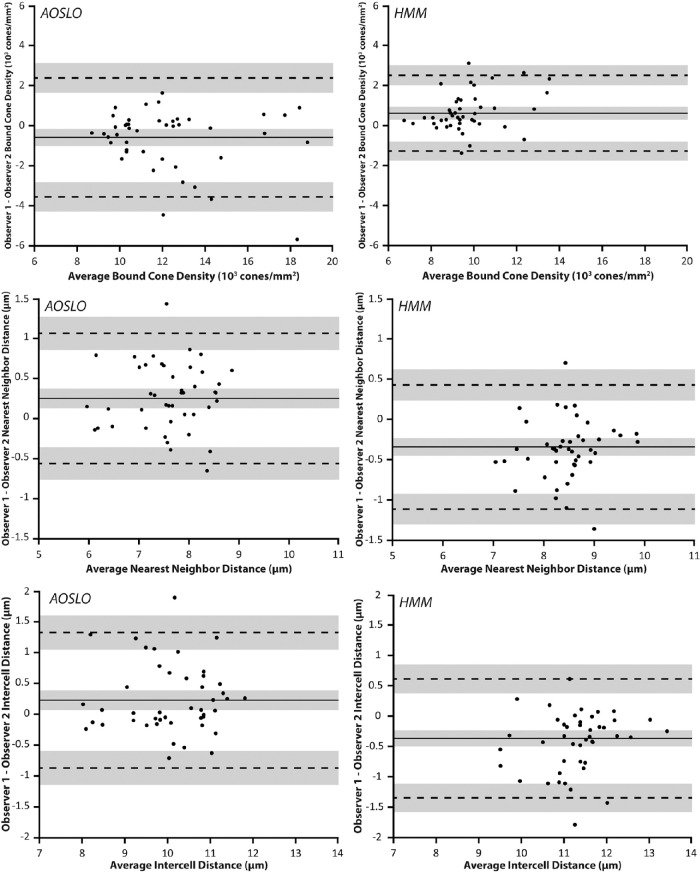
Bland–Altman plots of bound cone density, NND, and ICD for observer 1 and observer 2 on non-confocal AOSLO and HMM. *Solid lines* indicate the mean biases; *dashed lines* indicate the upper and lower limits of agreement. The *shaded areas* surrounding these lines indicate the 95% confidence intervals for these values.

For the HMM images, the intergrader ICC for cone density estimates was 0.739 (95% confidence interval [CI], 0.611–0.868), NND was 0.688 (95% CI, 0.538–0.838), and ICD was 0.757 (95% CI, 0.636–0.878). In AOSLO images, these values were 0.836 (95% CI, 0.750–0.921), 0.805 (95% CI, 0.705–0.905), and 0.835 (95% CI, 0.748–0.921), respectively. Interobserver Bland–Altman analysis showed a mean bias between observers one and two of 587 cones/mm^2^ for cell density, 0.25 µm for NND, and 0.22 µm for ICD on AOSLO images; for HMM images, the mean biases were slightly larger at 614 cones/mm^2^ for density, 0.34 µm for NND, and 0.33 µm for ICD (Fig. 4). The average value from the two observers was used for subsequent analyses.

**Figure 5.**
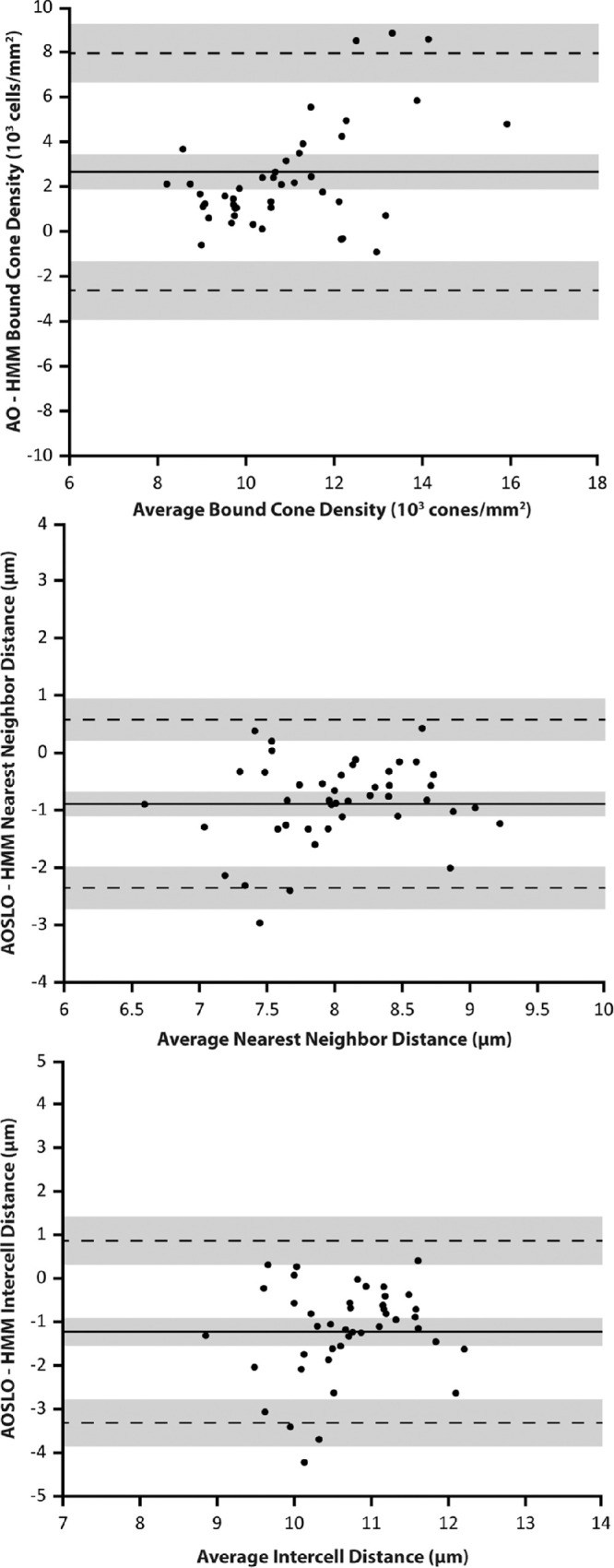
Bland–Altman plots demonstrating inter-device agreement (AOSLO and HMM). *Solid lines* indicate the mean biases; *dashed lines* indicate the upper and lower limits of agreement. The *shaded areas* surrounding these lines indicate the 95% confidence intervals for these values.

Averaged bound cone density measurements on AOSLO ranged from 8691 to 18,798 cones/mm^2^, with a mean of 12,375 cones/mm^2^; for HMM imaging, the range of averages was 6741 to 13,530 cones/mm^2^, with a mean of 9713 cones/mm^2^. Averaged NND measurements for AOSLO ranged from 5.97 to 8.86 µm with a mean of 7.56 µm; average NND measurements from HMM images ranged from 7.05 to 9.86 µm with a mean of 8.45 µm. Averaged ICD measurements on AOSLO ranged from 8.02 to 11.81 µm with a mean of 10.04 µm. The same measurements on HMM ranged from 9.51 to 13.42 µm with a mean of 11.27 µm. Bland–Altman analysis of inter-device differences showed a mean bias of 2661 cones/mm^2^ for density estimates, –0.89 µm for NND, and –1.23 µm for ICD between AOSLO and HMM (Fig. 5).

## Discussion

Although AOSLO devices remain expensive and have limited availability, commercially available devices such as the HMM have a role in contributing to our understanding of the photoreceptor mosaic in larger populations. This study aimed to assist in interpreting the results of quantitative studies on HMM, with reference to split-detection AOSLO, the gold standard for unambiguous cone identification outside the fovea. We found that, on average, the HMM underestimates cone density and overestimates cone spacing compared to AOSLO.

One of the most notable advantages of the HMM over AOSLO is the ease of image acquisition, although the reported success rates of HMM imaging are variable. Among the publications that have done so, figures range from 45% to 75% (Bartsch DUG, et al. *IOVS*. 2021;62:ARVO E-Abstract 24),^37^^,^^38^ lower than our success rate of 80%. Although this may be due to inclusion of a greater number of participants with disease in one study,^37^ even among participants with normal vision our success rate was higher. This may have been due to our use of one operator throughout (NW), which allowed for development of acquisition skills across the range of participant findings. The use of the high-speed modality and additional offline averaging combined with a lower number of ART frames is also likely to have contributed to the success rate. Although the high-resolution mode is likely to result in improved image quality for individuals with normal fixation and excellent ability to cooperate with the requirements of imaging, in our mixed cohort with and without retinal dysfunction (and nystagmus) we used the high-speed mode across all subjects to ensure that a good dataset was achievable for all. We believe this contributed to our higher success rate; however, our use of high-resolution mode may have resulted in a higher or lower success rate, and in the clinic the appropriate mode for each patient may be selected on a case-by-case basis. Acquisition of a higher number of images in individuals with poorer quality images likely increased the success rate compared to studies in which a fixed number of images (or even just a single image) was reported to be captured at each location. Nonetheless, all imaging was performed without refractive correction, which is proposed to further enhance image quality on HMM. However, the impact of refractive correction on image scale would be critical to confirm to ensure the accuracy of the quantitative metrics.^42^ As we avoid the use of refractive correction with our AOSLO imaging, we felt it important not to introduce additional scaling differences in our HMM images that were not present in the AOSLO images.

**Figure 6.**
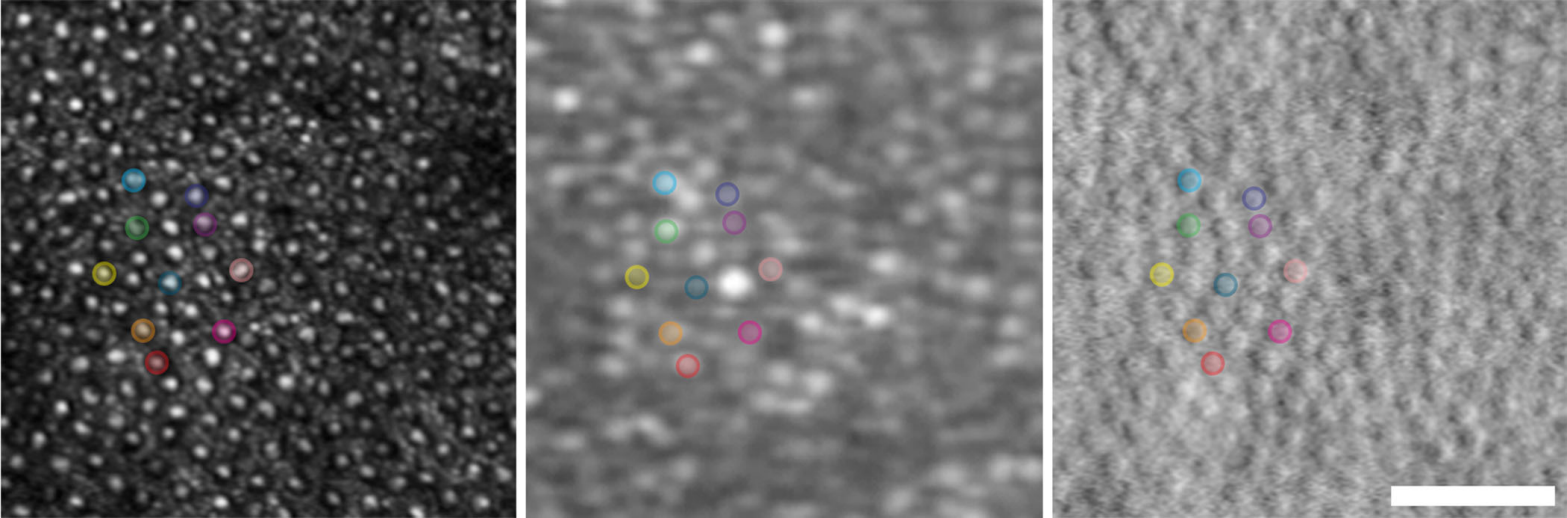
Inter-device image alignment. Images from one 23-year-old participant with normal vision demonstrate the cone-for-cone alignment possible with careful custom transformation: (A) confocal AOSLO; (B) HMM; and (C) split-detection AOSLO. Matching individual cones are highlighted across all three images. *Scale bar*: 50 µm. See Supplementary Video S2 for overlaid images.

We found density estimates of the same retinal location to be lower on HMM images when compared to nonconfocal AOSLO. One potential explanation for this is the approximate alignment method used. Garrioch et al.^50^ showed that exact alignment of ROIs in AOSLO studies improved the reliability of both density and spacing metrics of the cone mosaic. This was not possible for most ROIs, given the different overall appearance of the mosaic in the two modalities. However, our approach to co-localization of ROIs between modalities using large anatomical landmarks such as vessels prevented large misalignment of ROIs. Although cone packing density changes rapidly within the central 1° to 2°, we constrained our analysis to regions where cone density is more uniform; thus, small shifts in ROI placement between modalities are unlikely to account for all of the observed differences in density. Support for this is found in an analysis of one subject who had few higher order aberrations, resulting in HMM image quality that was sufficient to facilitate an exact cone-for-cone alignment between modalities using manual transformations in Photoshop (Fig. 6). The theoretical HMM scale (as calculated above) was found to be within 0.3% of the AOSLO scale in this ROI. The bound density (averaged from both graders) on AOSLO was 11,706 cones/mm^2^, and on HMM it was 11,318 cones/mm^2^. The average NNDs were 7.74 µm and 7.71 µm and the average ICDs were 10.17 µm and 10.37 µm on AOSLO and HMM, respectively. Additional explanations for the different cone mosaic metrics observed between the devices across all subjects could be errors in deriving the image scale in either modality. In HMM images, scaling errors may be contributed to by greater image distortions accounting for poorer resolution of the cone mosaic in subjects with more typical image quality. Lower resolution on the HMM may also lead to the appearance of neighboring cones as a single cone in these HMM images of more typical quality.

The known short-term variation in cone reflectivity may interfere with their visibility on HMM imaging. Given the dependence of HMM image quality on whole-frame averaging achieved with the ART mode, significant short-term changes in the reflectivity of one cone in relation to its neighbors across frames could be contributing to registration errors and poorer distinction of adjacent structures. Selection of raw frames in which varying cone intensity is seen through postprocessing and the use of a maximum-intensity projection instead of averaging may help to combat this, but this approach was not explored as part of the current study.

One previously published study directly comparing densities between confocal AOSLO and HMM reported photoreceptor densities using the HMM within 10% of densities in the same region with confocal AOSLO (Bartsch DUG, et al. *IOVS*. 2021;62:ARVO E-Abstract 24). Our data show an average underestimation on HMM of 24% compared to nonconfocal AOSLO, with a range of 97% underestimation to 8% overestimation. The previous study did not report the range of eccentricities from which the AOSLO and HMM densities were compared, but data presented here cover 3.0° to 10.0° eccentricity superiorly and 2.9° to 9.1° temporally. There were statistically significant associations between the difference in density estimate and eccentricity (*P* < 0.0001), the difference in NND and eccentricity (*P* = 0.0010), and the difference in ICD and eccentricity (*P* = 0.0002); although these associations were weak (*r*^2^ = 0.40, 0.21, and 0.26, respectively), higher eccentricities were associated with greater agreement. The deviation from previously reported figures likely reflects a different range of eccentricities and therefore a greater range of densities included, although it also supports the idea that small misalignments in these higher density areas may have contributed to the greater disagreement between modalities, as the cone-for-cone alignment demonstrated in Figure 6 was not possible across all subjects.

Our data show higher average intergrader agreement on nonconfocal AOSLO than HMM images, although confidence intervals overlap. This may be explained by the principles underlying split-detection and HMM imaging modalities. HMM imaging has a similar appearance to confocal AOSLO, which depends on the waveguiding of the photoreceptor. In the parafovea, larger cones have less uniform intensity profiles caused by passing of higher waveguide modes,^54^ which can make them more challenging to distinguish from the surrounding rods.^55^ In contrast, split-detection images are not thought to rely on waveguiding, as split-detection imaging in patients with non-waveguiding cones reveal inner segment structures.^8^^,^^56^ This accounts for known superior intergrader agreement in split-detection than confocal AOSLO images outside the macula.^8^^,^^52^ It is worth considering that our intergrader ICCs are lower than those reported by Cunefare et al.,^57^ which may be explained by the fact that 85% of images included in the current study were captured at 1.5° field of view, compared to images exclusively captured at 1° by Cunefare et al. Our intergrader ICC for cone density on HMM was much higher than that previously reported by Mendonça et al.^38^ Their low ICC of 0.22 is likely to be secondary to differing internal rules for cone identification. Naïve graders of retinal images with single-cell resolution have been shown to have measurably lower repeatability^55^; thus, the prior experience of both of our graders with analyzing confocal AOSLO images is likely to have contributed to our higher ICC of 0.739 (95% CI, 0.611–0.868). Although ICC values comparable to ours with overlapping 95% confidence intervals have been reported in studies with graders inexperienced with AOSLO imaging (0.891 (95% CI, 0.696–0.952), these values were based on raw cone counts rather than bound cone density, as examined in our study.^39^ Interestingly, the intergrader ICC of our raw cone counts was higher at 0.921 (95% CI, 0.861–0.983), indicating interobserver variability in identification of cells at the edge of an ROI.

There were some limitations to this study. Firstly, we only examined individuals with contiguous mosaics. The topography of the cone mosaic and the appearance of individual photoreceptors can vary widely in confocal AOSLO images of patients with retinal degenerative conditions,^58^^–^^66^ and similar variability would be expected in HMM images. In conditions where cone waveguiding is impaired,^66^^–^^68^ unambiguous identification of cones in HMM images would be more challenging. In other cases where there is only decreased density of remnant cones, resolution on the HMM images might be easier and could show better agreement with AOSLO-based measures. Additional studies including individuals with a range of retinal diseases will be required to understand the relationship between photoreceptor metrics for AOSLO and HMM in the diseased retina. Another consideration is that some participants had HMM imaging performed up to 18 months after AOSLO, although we would expect little change in cone structure over this time frame.^69^ Accordingly, linear regression revealed no association between the time elapsed between imaging modalities and the difference in cone density (*r*^2^ = 0.003; *P* = 0.72), NND (*r*^2^ = 0.002; *P* = 0.78), or ICD (*r*^2^ = 0.002; *P* = 0.77). Thus, although we would ideally want the HMM and AOSLO images to be collected on the same day in all participants, this difference is unlikely to account for the lower estimates of density on HMM in our participants. Finally, the offline averaging step we utilized in the processing of HMM images enhanced the resolvability of the individual photoreceptors (Fig. 2). This processing was not as time consuming and labor intensive as the processing required for AOSLO images and was undertaken in an effort to maximize the quality of HMM images for comparison to AOSLO. For individuals with excellent optical quality and minimal aberrations, this averaging step may not be necessary to extract reasonable, analyzable images of the mosaic; however, it may be required to extract useful quantitative information in those with suboptimal image quality.

Despite these limitations to quantitative assessment of the photoreceptor mosaic with the HMM imaging system, its commercial availability has greatly expanded the ability for clinicians to non-invasively image the human retina with cellular resolution. As the resolution of the HMM differs from that achieved with more complex and expensive AO-based devices, direct comparison between these modalities is needed to define the capabilities of the HMM in various clinical populations. We have shown that cone density is lower and spacing estimates are greater on HMM compared to nonconfocal AOSLO. There are known differences in cone metrics when using confocal AOSLO versus nonconfocal AOSLO,^52^^,^^55^ so it would be interesting to equip the SPECTRALIS HMM with a split-detection channel to isolate the contribution of the device in future studies. Although clinical populations commonly have such higher order aberrations, which would likely result in limited resolution of higher density regions, HMM may still prove useful for gauging photoreceptor numerosity in more eccentric locations within contiguous mosaics. With an understanding of the tendency of HMM images to underestimate density by comparison to nonconfocal AOSLO, it may become a complementary tool to help screen patients in clinical settings to be referred for more extensive AO-based imaging.

## Supplementary Material

Supplement 1

Supplement 2

Supplement 3
