# Supplementary material for: Comparison of Cone Mosaic Metrics From Images Acquired With the SPECTRALIS High Magnification Module and Adaptive Optics Scanning Light Ophthalmoscopy
Source: Transl Vis Sci Technol. 2022 May 18;11(5):19. doi: 10.1167/tvst.11.5.19 (PMC9123519; doi:10.1167/tvst.11.5.19)
Supplement: Supplement 1 [file tvst-11-5-19_s001.pdf]

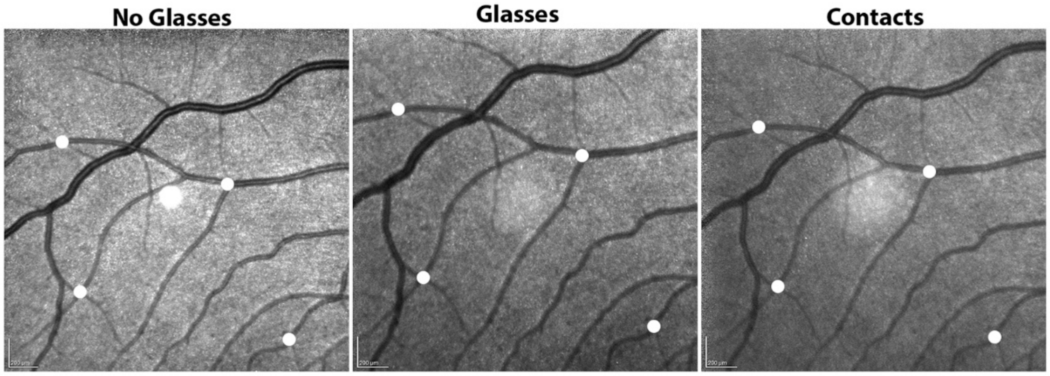

Supplementary Figure S1 - Scale differences observable with corrective lenses. Successive images of the same retina acquired using no refractive correction, glasses, and contacts. Note the image quality with the contacts is somewhat degraded due to tear film issues; but large features are still resolvable. The scale bar provided by Spectralis is identical in all three images, however, the image acquired with glasses is magnified by 12% and the image acquired with contacts is magnified by 4.5%. Four common points are shown from which the relative scaling between the images was computed.
